# Supplementary material for: Isolation Procedure for CP E. coli from Caeca Samples under Review towards an Increased Sensitivity
Source: Microorganisms. 2021 May 20;9(5):1105. doi: 10.3390/microorganisms9051105 (PMC8161246; doi:10.3390/microorganisms9051105)
Supplement: Supplementary file 1 [file microorganisms-09-01105-s001.zip › microorganisms-1184957-supplementary.pdf]

Supplemental Material Table 1: Detailed information about the used CPE. The seven CPE used for pre-studies are highlighted in grey.
Ampicillin (AMP); Azithromycin (AZI); Cefepime (FEP); Chloramphenicol (CHL); Ciprofloxacin (CIP); Colistin (COL); Ertapenem (ERP); Cefotaxime (FOT); Cefoxitin (FOX); Gentamicin (GEN); Imipenem (IMI); Kanamycin (KAN); Meropenem (MERO); Nalidixic acid (NAL); Sulfamethoxazole (SMX); Ceftazidime (TAZ); Tetracycline (TET); Tigecycline (TGC); Trimethophrim (TMP).

**Isolate no.**	**Carbapenemase**	**AMP**	**AZI**	**CEF**	**CHL**	**CIP**	**COL**	**ERP**	**FOT**	**FOX**	**GEN**	**IMI**	**MERO**	**NAL**	**SMX**	**TAZ**	**TET**	**TGC**	**TMP**
CP_1	GES-5	>64	4	0.25	≤8	0.03	≤1	0.25	64	32	2	0.5	0.12	≤4	>1024	8	64	≤0.25	>2
CP_2	KPC-2	>64	8	32	>128	>8	≤1	>2	>64	>64	>32	4	8	>128	≤8	32	>64	≤0.25	≤0.25
CP_3	GES-5	>64	4	0.5	≤8	≤0.015	≤1	0.25	2	64	2	0.5	0.25	≤4	>1024	8	64	≤0.25	>32
CP_4	KPC-2	>64	>64	8	16	>8	≤1	>2	>16	16	32	4	4	>128	>1024	16	>64	≤0.25	32
CP_5	NDM-1	>64	16	>32	>128	>8	≤1	>2	>64	>64	>32	>16	>16	>128	>1024	>128	>64	≤0.25	>32
CP_6	OXA-48	>64	4	0.5	≤8	0.03	≤1	2	1	8	≤0. 5	1	1	≤4	≤8	≤0.5	≤2	≤0.25	≤0.25
CP_7	OXA-48	>64	8	1	16	0.12	≤1	>2	4	16	≤0.5	2	2	≤4	>1024	8	4	≤0.25	1
CP_8	VIM-1	>64	64	>32	>128	0.5	≤1	2	>64	>64	2	4	4	16	>1024	>128	≤2	0.5	>32
CP_9	VIM-1	>64	8	16	>128	≤0.015	≤1	0.5	32	32	2	4	2	≤4	>1024	128	64	0.5	≤0.25
CP_10	VIM-1	>64	8	4	128	≤0.015	≤1	0.12	32	16	1	2	0.25	≤4	>1024	>8	≤2	≤0.25	32
CP_11	VIM-1	>64	8	16	128	≤0.015	≤1	0.12	>64	32	1	4	1	≤4	16	64	4	0.5	>32
CP_12	VIM-1	>64	16	16	>8	≤1	>2	>32	>64	>64	32	8	8	>128	>1024	>8	4	≤0.25	>32
E. coli	VIM-1	>64	8	16	64	0.03	≤1	0.12	>64	32	1	4	1	≤4	32	64	4	0.5	>32
S. Corvallis	NDM-1	>64	64	16	>128	0.5	4	>2	>64	>64	1	4	8	32	>1024	>64	64	0.5	≤0.25
S. Infantis	VIM-1	>64	8	>32	>128	≤0.015	≤1	1	>64	64	1	4	4	≤4	>1024	>128	≤2	0.5	>32
S. Infantis	VIM-1	>64	8	>32	>128	≤0.015	≤1	0.5	>64	>64	1	4	4	≤4	>1024	>128	≤2	0.5	>32

Supplemental Material Table 2: The repeated isolation of CPE from the spiked faecal samples. Method A is the EURL-method. Method B is the modified method. For calculation, the OXA and CARBA- site of the chromID ® SMART CARBA agars and of the McC+CTX+MEM and the McC+MEM plates of the *in-house* agar were combined. „+" described the confirmed positive samples and "-" the confirmed negative samples..

	Week 1					Week 2					Week 3				
Sample no.	Spiked isolate	Method A + chromID ® SMART CARBA agars	Method A + *in-house* agars	Method B + *in-house* agars	Method B + chromID ® SMART CARBA agars	Spiked isolate	Method A + chromID ® SMART CARBA agars	Method A + *in-house* agars	Method B + *in-house* agars	Method B + chromID ® SMART CARBA agars	Spiked isolate	Method A + chromID ® SMART CARBA agars	Method A + *in-house* agars	Method B + *in-house* agars	Method B + chromID ® SMART CARBA agars
1	CP_1	-	+	+	-	ATCC25922	-	-	-	-	ATCC25922	-	-	-	-
2	CP_2	+	+	+	+	CP_1	- -	+	-	-	CP_1	+	+	-	-
3	CP_3	+	+	-	-	CP_2	+	+	+	+	CP_2	+	+	+	+
4	CP_4	+	+	+	+	CP_3	+	+	.	-	CP_3	+	+	-	-
5	ATCC25922	-	-	-	-	ATCC25922	-	-	-	-	ATCC25922	-	-	-	-
6	ATCC25922	-	-	-	-	CP_4	+	+	+	+	CP_4	+	+	+	+
7	CP_5	+	+	+	+	CP_5	+	+	+	+	CP_5	+	+	+	+
8	CP_6	+	+	+	+	ATCC25922	-	-	-	-	ATCC25922	-	-	-	-
9	CP_7	+	+	+	+	ATCC25922	-	-	-	-	ATCC25922	-	-	-	-
10	CP_8	+	+	+	+	ATCC25922	-	-	-	-	ATCC25922	-	-	-	-
11	ATCC25922	-	-	-	-	CP_7	+	+	+	+	CP_7	+	+	+	+
12	CP_9	+	+	+	+	CP_8	+	+	+	+	CP_8	+	+	+	+
13	ATCC25922	-	-	-	-	CP_9	+	+	+	+	CP_9	+	+	+	+
14	ATCC25922	-	-	-	-	CP_10	-	+	+	+	CP_10	+	+	+	+
15	CP_10	-	+	+	-	CP_11	-	+	+	-	CP_11	-	+	+	-
16	CP_11	-	+	+	-	ATCC25922	-	-	-	-	ATCC25922	-	-	-	-
17	CP_12	+	+	+	+	CP_12	-	+	+	-	CP_12	-	+	+	-
18	ATCC25922	-	-	-	-	CP_13	+	+	+	+	CP_13	+	+	+	+

Supplemental Material Table 3: Summarized real-time PCR results on the repeated detection of carbapenemase genes from the spiked faecal samples.

	Week 1					Week 2					Week 3				
Sample no.	Carba-penemase	BPW	LB+CTX	LB+MEM	Both LB-enrichements	Carba-penemase	BPW	LB+CTX	LB+MEM	Both LB-enrichements	Carba-penemase	BPW	LB+CTX	LB+MEM	Both LB-enrichements
1	GES	+	-	-	-	ATCC25922	-	-	-	-	ATCC25922	-	-	-	-
2	KPC	+	+	+	+	GES	+	+	-	+	GES	+	+	-	+
3	GES	+	+	-	+	KPC	+	+	+	+	KPC	+	+	+	+
4	KPC	-	+	-	+	GES	+	+	+	+	GES	+	+	+	+
5	ATCC25922	-	-	-	-	ATCC25922	-	-	-	-	ATCC25922	-	-	-	-
6	ATCC25922	+ (GES)	-	-	-	KPC	-	+	+	+	KPC	-	+	+	+
7	NDM	+	+	+	+	NDM	+	-	+	+	NDM	+	+	+	+
8	OXA	+	-	+	+	ATCC25922	-	-	-	-	ATCC25922	-	-	-	-
9	OXA	+	+	+	+	ATCC25922	-	-	-	-	ATCC25922	-	-	-	-
10	VIM	-	+	-	+	ATCC25922	-	-	-	-	ATCC25922	-	+	-	+
11	ATCC25922	-	-	-	-	OXA	+	-	+	+	OXA	+	-	+	+
12	VIM	-	+	-	+	OXA	+	+	+	+	OXA	+	+	+	+
13	ATCC25922	-	-	-	-	VIM	+	+	+	+	VIM	+	+	+	+
14	ATCC25922	-	-	-	-	VIM	-	+	-	+	VIM	-	+	-	+
15	VIM	-	+	-	+	VIM	-	+	-	+	VIM	-	+	-	+
16	VIM	-	-	-	-	ATCC25922	-	-	-	-	ATCC25922	-	-	-	-
17	VIM	+	+	+	+	VIM	-	+	-	+	VIM	-	+	-	+
18	ATCC25922	-	-	-	-	VIM	-	+	+	+	VIM	+	+	+	+
